# Performance and Cost-Effectiveness of Tumor Tissue-Modified Viral (TTMV)-Human Papillomavirus (HPV) DNA for Surveillance of HPV-Associated Oropharyngeal Cancer Compared With Standard Positron Emission Tomography (PET)

**DOI:** 10.7759/cureus.112879

**Published:** 2026-07-17

**Authors:** Phoebus Sun Cao, Rui Song, Karna T Sura

**Affiliations:** 1 Radiation Oncology, SUNY Upstate Medical University, Syracuse, USA

**Keywords:** cost-benefit analysis, cost-effective cancer care, head neck cancer, hpv-associated cancers, markov decision process, navdx, pet scans

## Abstract

Introduction: The standard of care for surveillance of human papillomavirus (HPV)-associated oropharyngeal cancer squamous cell carcinoma (OPSCC) is post-treatment positron emission tomography/computed tomography (PET/CT) imaging. There has been increasing interest in utilizing plasma tumor tissue-modified viral (TTMV)-HPV DNA as part of the diagnostic and surveillance paradigm.

Methodology: In this single-institution retrospective analysis, we assessed the performance and cost-effectiveness of surveillance TTMV-HPV DNA in HPV-associated OPSCC among patients who underwent definitive or adjuvant radiation treatment. Patients included in our analysis underwent a three-month post-treatment PET scan and three-month TTMV-HPV DNA testing, with subsequent testing per the provider's discretion at follow-up. Markov decision tree analysis was performed to evaluate the cost-benefit of using TTMV-HPV DNA testing as part of the surveillance paradigm, particularly when a PET/CT image was deemed equivocal.

Results: From 2018 to 2023, 68 patients with HPV-associated OPSCC underwent post-treatment surveillance with PET/CT and TTMV-HPV DNA testing. For PET/CT surveillance, the sensitivity, specificity, positive predictive value (PPV), and negative predictive value (NPV) were 83.3%, 90.3%, 45.5%, and 98.2%, respectively. For surveillance with TTMV-HPV DNA, the sensitivity, specificity, PPV, and NPV were 66.7%, 98.4%, 80.0%, and 96.8%, respectively. Using Markov decision tree analysis, we assessed the net monetary benefit (NMB) of additional TTMV-HPV DNA testing when PET/CT was equivocal and the NMB of upfront TTMV-HPV DNA testing alongside PET/CT. In both scenarios, we noted that the NMB was greater with TTMV-HPV DNA (scenario 1: $16,925; scenario 2: $16,809) than with repeat PET/CT (scenario 1: $15,575; scenario 2: $15,504).

Conclusions: These findings suggest that TTMV-HPV DNA testing performs as well as, and is potentially more cost-effective than, gold-standard PET/CT imaging for the surveillance of HPV-associated OPSCC. Future randomized studies are needed to determine whether TTMV-HPV DNA testing alone could be sufficient for the surveillance of treated HPV-associated OPSCC.

## Introduction

In the United States, the incidence of human papillomavirus (HPV)-associated oropharyngeal squamous cell carcinoma (OPSCC) continues to rise and accounts for 70%-80% of new cases [1,2].​ This trend has also been observed in conjunction with an overall decrease in non-HPV-associated OPSCC [3,4].​ While the rising incidence tends to skew toward younger individuals and males, rates are also rising among older patients who were previously exposed [5].​ Nonetheless, in multiple studies, patients with HPV-associated OPSCC have been found to have improved overall survival outcomes across age groups and regardless of treatment modality [4,5].

While there are over 100 strains of HPV, a meta-analysis noted that 80%-90% of HPV-associated OPSCC can be attributed to the p16 subtype, with other subtypes such as HPV 18, 31, and 33 also being common [6].​ Thus, there has been great interest in identifying strategies to reliably detect HPV-associated OPSCC at diagnosis and at recurrence. While Southern Blot was historically the gold standard for detection, it is technically demanding and is seldom used in modern clinical practice except to confirm other HPV detection methods. Instead, tumor tissue-modified HPV DNA (TTMV-HPV DNA) assays represent a clinically validated method for detecting circulating tumor HPV DNA (ctHPV DNA) in plasma [6, 7].​ At this institution, we utilize TTMV-HPV DNA testing (NavDx®, Naveris, Waltham, MA).

Positron emission tomography/computed tomography (PET/CT) imaging is currently the standard of care for staging and assessing treatment response. Meta-analyses have supported the use of PET/CT as an essential component for the initial staging and post-treatment surveillance of head and neck cancers [8,9]. ​Particularly for disease recurrence, the positive predictive value (PPV) and negative predictive value (NPV) of PET/CT were found to be 96.3% and 88.3%, respectively [9].​ However, other studies have found the PPV of PET/CT can be as low as 60%-70% [10,11].​ When a PET/CT is equivocal, tissue sampling is often indicated; however, this approach has its own limitations, with fine needle aspiration (FNA) biopsy demonstrating a diagnostic accuracy of 70%-80% [12]. Additionally, PET/CT is typically performed only at three months post-treatment as a baseline, leaving clinical examination and nasopharyngolaryngoscopy (NPL) as the mainstays of surveillance thereafter.

It is also noted that the costs associated with the treatment of head and neck cancers can be substantial just in the treatment phase alone. One prior report on oral cavity, oropharynx, and salivary gland cancers reported that the cost for a course of definitive chemoradiation (CRT) can range from as low as $85,462 with Medicare to as high as $137,315 with commercial insurance, with the costs certainly rising since that time [13].​ The process of undergoing routine surveillance imaging after definitive CRT not only adds additional costs to overall care but can also give additional stress in the setting of equivocal findings that may indicate invasive testing. 

In this retrospective analysis of patients with HPV-associated OPSCC who received definitive CRT, we sought to assess the effectiveness of TTMV-HPV DNA in the follow-up setting compared with standard PET/CT imaging, both as a surveillance tool and as a cost-effective strategy that could potentially reduce repeat imaging and invasive testing.

## Materials and methods

Data collection and analysis 

A retrospective, IRB-exempt study (2088135-2) was conducted at our institution. A chart review was conducted to identify all patients with HPV-associated OPSCC treated with definitive CRT, definitive radiation therapy (RT), or surgery followed by postoperative radiation between 2018 and 2023 at a single institution. All patients received all aspects of treatment at this single institution. All patients were retrospectively confirmed to have received standard-of-care treatment. Patient characteristics, including patient age, gender, and disease stage, were obtained from the clinical chart. Radiation treatment details and plan, including radiation dose, were obtained from the treatment planning system. All patients underwent PET/CT imaging and TTMV-HPV DNA testing approximately 3 months after treatment completion. Patients who were lost to follow-up or had incomplete surveillance imaging or testing were removed from the analysis. Additional TTMV-HPV DNA testing, imaging, and biopsies were ordered based on provider preference. Risk score interpretation of TTMV-HPV DNA is summarized in Table 1 [14].​ Imaging response on PET/CT was assessed using the Neck Imaging Reporting and Data System (NI-RADS) risk classification [14]. PET/CT reports that did not detail standardized uptake values (SUVs) were individually evaluated with measurements compared to blood pool and liver activity being performed. Calculations for median radiation and TTMV-HPV DNA values, as well as sensitivity, specificity, PPVs/NPVs for either PET/CT imaging or TTMV-HPV DNA testing, were performed in Microsoft Excel. Confidence intervals for sensitivity and specificity were generated using exact Clopper-Pearson confidence intervals. Confidence intervals for predictive values were generated using standard logit confidence intervals. McNemar's test was performed to compare the sensitivities and specificities of each modality, assuming an alpha significance of 0.05.

**Table 1:** Risk score interpretation of TTMV-HPV DNA results. HPV, human papillomavirus; TTMV-HPV DNA, tumor tissue-modified viral-HPV DNA

TTMV-HPV-16 DNA score	TTMV-HPV-18, -31, -33, -35 DNA score	Risk score interpretation
<2	<5	Negative
2-3	5-12	Indeterminate
>3	>12	Positive

Decision tree 

We compared the cost-effectiveness of post-treatment TTMV-HPV DNA and PET/CT for patients with HPV-associated OPSCC who received standard CRT under two clinical scenarios. In the first scenario, repeat PET/CT was compared with TTMV-HPV DNA testing after an equivocal three-month post-treatment PET/CT. The second scenario directly compared TTMV-HPV DNA with PET/CT as the test of choice at 3 months post-treatment. For these two scenarios, we constructed Markov decision models using TreeAge Pro 2023 (TreeAge Software, LLC, Williamstown, MA) to simulate post-treatment surveillance. This type of analysis utilizes the probability of each test outcome, along with the respective costs, to determine which testing strategy is most cost-effective at a predetermined willingness-to-pay threshold. The test outcome probabilities were extrapolated from our retrospective analysis, particularly the probabilities of positive/negative results for TTMV-HPV DNA and positive/equivocal/negative results for PET/CT. Equivocal PET/CT was defined as a NI-RADS score of 2 at the three-month follow-up after completion of definitive CRT. Cost-benefit was evaluated using net monetary benefit (NMB), which incorporates both costs and health benefits (QALYs). All external model inputs are summarized in Table 2. The costs of TTMV-HPV DNA testing and PET/CT imaging were obtained from previously published literature. The estimated recurrence rate was extrapolated from a median time to recurrence of 8.2 months based on a combined retrospective analysis of RTOG 0129 and RTOG 0522 [15]. As the decision model starts at the first three-month follow-up, when both PET/CT and TTMV-HPV DNA would be available for interpretation, each cycle was three months in length, with a time horizon of one year. Health states for PET/CT included positive, negative, or equivocal results, with subsequent decisions to undergo biopsy, continued follow-up, or TTMV-HPV DNA testing. Health states for TTMV-HPV DNA testing included positive or negative results, with subsequent decisions to undergo continued observation or diagnostic PET/CT.

**Table 2:** Model inputs for Markov assessment. Estimates for the costs of TTMV-HPV DNA testing and PET/CT were drawn from Lin et al. [16,17]. Estimates of progression-free survival, life-years, and recurrence were obtained from Fakhry et al. [15]. TTMV-HPV, tumor tissue-modified viral-human papillomavirus; PET/CT, positron emission tomography/computed tomography

Variable	Value	Source
Recurrence (yearly)	0.683	Fakhry et al. [15]
TTMV-HPV DNA ($)	150	Lin et al. [16]
PET/CT ($)	1,500	Lin et al. [17]

## Results

Patient characteristics 

Our cohort consisted of 68 patients, predominantly male (60, 88.2%), with a median age of 66 years. Half of the cohort (34, 50.0%) were never smokers. AJCC 8th edition cancer staging was as follows: Stage I (27, 39.7%), Stage II (31, 45.6%), Stage III (7, 10.3%), and Stage IV (3, 4.4%). The majority (64, 94.1%) of patients received definitive CRT, with 95.6% of these patients receiving a radiation dose of 70 Gy. A total of 179 TTMV-HPV DNA tests and 88 PET scans were obtained. Pre-treatment TTMV-HPV DNA was available for 41 patients (60.3%); the median positive TTMV-HPV DNA value at initial diagnosis was 470.5. Six patients (8.8%) experienced disease failure; in three of these patients, TTMV-HPV DNA indicated failure before imaging. The median time to positive TTMV-HPV DNA after treatment was 267 days, while the median time to positive imaging after treatment was 277 days; the median TTMV-HPV DNA value at recurrence was much lower than at initial diagnosis (28.5). At the time of data analysis, the median follow-up after treatment was 15.9 months. These findings are summarized in Table 3.

**Table 3:** Patient characteristics. TTMV-HPV, tumor tissue-modified viral-human papillomavirus; Gy, Gray; d, day

Characteristics	Values
Median age (years)	66
Sex (M), *n* (%)	60 (88.2%)
Smoking history (Yes), *n* (%)	34 (50.0%)
Primary tumor site, *n* (%)	
Base of tongue (BOT)	33 (48.5%)
Tonsil	30 (44.1%)
Others/Unknown primary	5 (7.4%)
Stage, *n* (%)	
I	27 (39.7%)
II	31 (45.6%)
III	7 (10.3%)
IV	3 (4.4%)
T stage, *n* (%)	
1	16 (23.5%)
2	21 (30.9%)
3	24 (35.3%)
4	5 (7.4%)
N stage, *n* (%)	
0	5 (7.4%)
1	36 (52.9%)
2	25 (36.8%)
3	2 (2.9%)
Concurrent chemotherapy, *n* (%)	64 (94.1%)
RT dose, *n* (%)	
70 Gy	65 (95.6%)
66 Gy	3 (4.4%)
Median RT dose (Gy)	70
Median TTMV-HPV DNA value at initial diagnosis	470.5
Recurrence, *n* (%)	6 (8.8%)
Time to positive imaging (d)	277
Time to positive biopsy (d)	302
Time to positive TTMV-HPV DNA (d)	267
Median TTMV-HPV DNA value at recurrence	28.5

In this analysis, NI-RADS assessment of PET/CT scans was found to have a sensitivity, specificity, PPV, and NPV of 83.3% (95% CI, 35.88%-99.58%), 90.3% (95% CI, 80.12%-96.37%), 45.5% (95% CI, 26.45%-65.88%), and 98.2% (95% CI, 90.33%-99.70%), respectively. Conversely, TTMV-HPV DNA was found to have a sensitivity, specificity, PPV, and NPV of 66.7% (95% CI, 22.28%-95.67%), 98.4% (95% CI, 91.34%-99.96%), 80.0% (95% CI, 34.56%-96.80%), and 96.8% (95% CI, 90.77%-98.95%). These findings are summarized in Table 4.

**Table 4:** Sensitivity, specificity, PPV, and NPV of PET imaging and TTMV-HPV DNA for detecting recurrence in p16-positive head and neck SCC treated with definitive or adjuvant radiation. NI-RADS, Neck Imaging Reporting and Data System; TTMV-HPV, tumor tissue-modified viral-human papillomavirus; CI, confidence interval; PPV, positive predictive value; NPV, negative predictive value; PET, positron emission tomography

NI-RADS (PET)			
Sensitivity	83.3% (95% CI, 35.88%-99.58%)	PPV	45.5% (95% CI, 26.45-65.88%)
Specificity	90.3% (95% CI, 80.12%-96.37%)	NPV	98.2% (95% CI, 90.33%-99.70%)
TTMV-HPV DNA			
Sensitivity	66.7% (95% CI, 22.28%-95.67%)	PPV	80.0% (95% CI, 34.56%-96.80%)
Specificity	98.4% (95% CI, 91.34%-99.96%)	NPV	96.8% (95% CI, 90.77%-98.95%)

When performing McNemar's test to assess the sensitivities of PET/CT and TTMV-HPV DNA testing for identifying recurrence, χ² = 0.3333 (*P* = 0.5637). When performing McNemar's test to assess the specificities of PET/CT and TTMV-HPV DNA testing for ruling out recurrence, χ² = 3.5714 (*P* = 0.0587).

Decision tree

**Figure 1:**
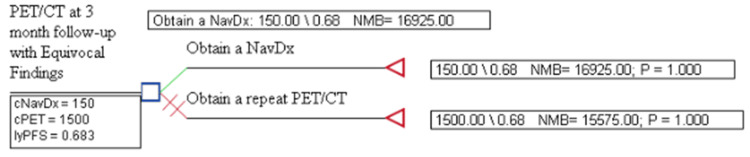
Cost-benefit decision tree comparing follow-up TTMV-HPV DNA testing with repeat PET after an equivocal three-month post-treatment PET. Created by authors using TreeAge Pro 2023 (TreeAge Software, LLC, Williamstown, MA). NMB, net monetary benefit; lyPFS, progression-free survival life-years; PET, positron emission tomography; NavDx, brand name for the TTMV-HPV DNA test; TTMV-HPV, tumor tissue-modified viral-human papillomavirus; NMB, net monetary benefit

In the first scenario, a Markov decision tree was built to assess the net monetary benefit (NMB) of performing TTMV-HPV DNA testing or repeat PET in the setting of an equivocal 3-month post-treatment PET/CT. In this setting, the NMB of follow-up TTMV-HPV DNA testing (NMB = $16,925) was greater than that of repeat PET (NMB = $15,575), as summarized in Figure 1. 

**Figure 2:**
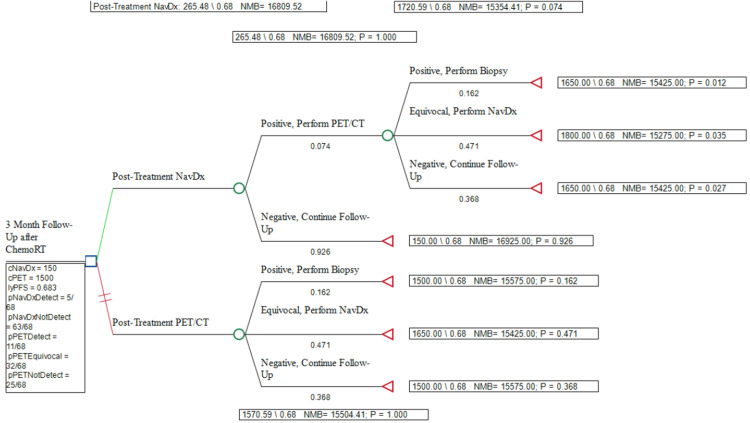
Cost-benefit decision tree comparing TTMV-HPV DNA with PET at the initial three-month follow-up following definitive non-operative treatment. Created by authors using TreeAge Pro 2023 (TreeAge Software, LLC, Williamstown, MA). ChemoRT, chemoradiation; NMB, net monetary benefit; lyPFS, progression-free survival life-years; PET, positron emission tomography; NavDx, brand name for the TTMV-HPV DNA test; TTMV-HPV, tumor tissue-modified viral-human papillomavirus

In the second scenario, a Markov decision tree was built to assess the NMB of TTMV-HPV DNA testing or PET/CT as the test of choice three months after definitive radiation treatment. The decision tree would then branch out accordingly based on whether TTMV-HPV DNA testing was positive or negative and whether the PET/CT had a positive, equivocal, or negative result. Overall, we noted that the NMB was greatest with TTMV-HPV DNA testing (NMB = $16,809) when compared to PET/CT (NMB = $15,504). This is even with accounting for the possibility that a positive TTMV-HPV DNA would indicate a subsequent PET/CT to localize recurrent disease. The results are summarized in Figure 2.

## Discussion

In the surveillance phase, PET/CT imaging continues to be a critical component in evaluating for residual/recurrent disease after definitive management of head and neck cancer. However, while being the standard of care, PET is not without its flaws. The PPV of PET can be as low as 60%-70%, and it is utilized only once, three months after definitive treatment [​10,11].​ Routine clinical surveillance thereafter has a dismal 0.2% rate of catching recurrences in asymptomatic patients [18].​ And when PET is equivocal, further invasive tissue sampling is needed to confirm recurrence, which also has limited performance [12​].

For prostate cancer, it has been a mainstay to utilize post-treatment prostate-specific antigen (PSA) to assess for biochemical recurrence after definitive management [19].​ While there are imaging modalities that evaluate for recurrent/residual prostate cancer, such as prostate-specific membrane antigen (PSMA) PET, these are performed upon suspicion for disease based on clinical evidence, including PSA increase, to help localize disease [20].​ In comparison, there is substantial potential heterogeneity for HPV-associated OPSCC due to the numerous strains of HPV present. However, given advances in immunohistochemistry (IHC) and TTMV-HPV DNA testing, there are effective ways to identify p16+ specific causes of HPV-associated OPSCC, which remains the most common subtype in 80%-90% of cases [​6].​ 

An initial retrospective study investigating TTMV-HPV DNA noted surveillance sensitivity and specificity of 79.2% and 100%, respectively. In addition, in this study, the PPV and NPV were found to be 100% and 98.2%, respectively [21].​ Recent large retrospective series and smaller prospective trials have corroborated the excellent sensitivity (88%-92%), specificity (up to 100%), PPV (up to 100%), and NPV (98%-100%) of TTMV-HPV DNA [22-24].​ In our analysis, TTMV-HPV DNA was found to have similar values of specificity, PPV, and NPV. This adds to the growing literature that TTMV-HPV DNA is a good test for recurrence of treated HPV-associated oropharyngeal cancer, along with standard post-treatment PET/CT. Through our McNemar's test, although the overall sensitivity of TTMV-HPV DNA appeared to be lower than expected, there is no statistical difference between the TTMV-HPV DNA and PET/CT for either sensitivity or specificity. Additionally, our study found that positive TTMV-HPV DNA predated imaging findings in half of the patients with disease recurrence. During the period of this study, when TTMV-HPV DNA testing was being implemented at our institution, there was no standardized timeframe for obtaining post-treatment TTMV-HPV DNA. Given the inherent ability of TTMV-HPV DNA to pick up recurrent disease at levels on average more than 15x lower than that at initial diagnosis, with standardized, routine implementation, we believe the effect seen here would be magnified. We have started a process to standardize the timeframe for TTMV-HPV DNA at our institution, given our findings and those of others on early recurrence detection with this test [22].​ 

While multiple studies have demonstrated the efficacy of TTMV-HPV DNA in the workup and surveillance setting of HPV-associated OPSCC, this is the first study that we are aware of utilizing a Markov decision tree to perform cost-benefit analysis comparing TTMV-HPV DNA against PET/CT, particularly at the initial three-month follow-up after definitive CRT. In our analysis, we noted that the NMB was higher both when performing a TTMV-HPV DNA after equivocal PET/CT at the three-month follow-up and when performing a TTMV-HPV DNA instead of standard PET/CT at the three-month follow-up. Our cost estimate for TTMV-HPV DNA was based on the self-pay option, which is around $150, but can be as low as $50 with financial assistance [​15].​ In addition, our estimate for the PET/CT was based on an estimated cost of $1,500, but it can be much higher, approaching $5,600, for self-pay options [15,16]. Consequently, when choosing TTMV-HPV DNA over PET/CT at the initial three-month follow-up, the NMB can likely be much greater, depending on the facility and location.

Limitations

There are several limitations of our analysis. Due to our small cohort, sensitivity and PPV were lower than prior estimates [22,23]. It should be noted, however, that the PPV and NPV of PET/CT in our study were 45.5% and 98.2%, respectively. This is higher compared with a UK retrospective review, which reported a PPV and NPV of 30% and 81.8%, respectively [25]. While this could be attributed to our small cohort, it nonetheless reaffirms concerns about routinely utilizing PET/CT as the primary method of disease surveillance. Critically, with only six recurrences out of 68 patients, relatively small differences can dramatically affect the sensitivity of either modality, as seen by values of 83% and 67% for PET/CT and TTMV-DNA, respectively, a significant limitation in a small cohort analysis and reflected in the wide confidence intervals. Moreover, since it was not initially routine to obtain a baseline TTMV-DNA before starting treatment, it is possible that some of the missed cases were in patients who would not have been detectable by TTMV-DNA even before treatment, thus undermining our sensitivity assessment. Nonetheless, in a clinical setting, a false positive PET/CT can contribute to greater distress, unnecessary procedures, and higher health costs overall, so additional complementary studies have value in aiding clinical decision making. Another limitation of our study is that during the period in which TTMV-HPV DNA was introduced, pre-treatment testing was not mandatory. While some studies have examined the diagnostic value of TTMV-HPV DNA, we are unable to comment on this or on how the magnitude of baseline testing affects performance in the surveillance setting [22]. Furthermore, while the focus of our analysis was the initial three-month post-treatment surveillance period, where both PET/CT and TTMV-HPV DNA testing are utilized, approximately 50% of failures occur by six months and 83% occur by two years, which are two timepoints beyond the scope of this analysis [26]. In these situations, routine PET/CT as surveillance would not be standard, but TTMV-HPV testing is a relevant tool for post-treatment detection. Nonetheless, the rate of TTMV-HPV testing had not been standardized at our institution and was left to the discretion of each respective physician, which represents another important limitation. Likewise, our Markov decision model analysis is flawed as it extrapolates the NMB of each decision pathway while utilizing a higher expected recurrence rate than what was found within our limited cohort. After the study period, the list price of the NavDx test was published as $2,200, which is much higher than the estimated price used in this analysis and represents another major source of limitation. In addition, our study was performed in the pre-immunotherapy time period. Finally, it is unclear how this may translate to future clinical practice in which maintenance immunotherapy may play a role, and how immunotherapy would affect the performance of this test.

## Conclusions

Our analysis adds to the growing body of evidence that TTMV-HPV DNA can be a reliable, accurate tool for surveillance of HPV-related head and neck cancers. In addition, there may be a greater NMB to performing a TTMV-HPV DNA in the setting of equivocal PET/CT imaging, and instead of a repeat PET/CT overall. While the small cohort and overall low rate of recurrence at short follow-up did hinder the sensitivity analysis of both PET/CT and TTMV-HPV DNA testing, there is an interesting question on whether TTMV-HPV DNA has the potential to be an alternative method of detection in the post-treatment surveillance setting, especially if the cost of PET/CT could be a limiting factor. These results should be considered exploratory as PET/CT continues to be the post-treatment standard at three-month follow-up. Future prospective randomized studies directly comparing TTMV-HPV DNA to PET/CT will be needed to determine if TTMV-HPV DNA alone on a routine schedule could be sufficient for surveillance of treated HPV-associated oropharyngeal cancer, especially if the overall cost of workup is a concern. PET/CT would remain an essential tool in interpreting equivocal lab findings or to help localize recurrent/residual disease.
